# Responsible Leadership Effect on Career Success: The Role of Work Engagement and Self-Enhancement Motives in the Education Sector

**DOI:** 10.3389/fpsyg.2022.888386

**Published:** 2022-04-27

**Authors:** Minyan Li, Feng Yang, Muhammad Waheed Akhtar

**Affiliations:** ^1^Department of Teacher Education, Taishan University, Taian, China; ^2^Department of Management Sciences, COMSATS University Islamabad, Islamabad, Pakistan

**Keywords:** responsible leadership, self-enhancement motives, work engagement, career success, education sector

## Abstract

Using social information processing theory, our study investigates the effect of responsible leadership on employee career success via work engagement. The model also examines whether self-enhancement motives moderate the aforementioned mediating linkages. In three waves, data were collected from employees in the education sector. Macro PROCESS was used to assess the hypotheses. According to the findings, responsible leadership boosts employee work engagement, which leads to career success. The results also suggest that responsible leadership has a stronger positive effect on work engagement among individuals high on self-enhancement motives. There is no evidence in the educational literature about the underlying process through which a responsible leadership impacts employee success. Our research addresses this gap by suggesting work engagement as a mediator of the effect of responsible leadership on individuals’ career success at various degrees of self-enhancement motives.

## Introduction

In today’s competitive environment, organizations develop internationally and confront several challenges to achieve their goals. Leadership is the most researched area in management since it is directly important in every aspect of the industry (Akhtar et al., 2021b). Educational leaders are required to play a variety of tasks, ranging from educational visionaries to legal overseers (Bartoletti and Connelly, 2013), highlighting the complexity of leadership in the education sector. Previous research has demonstrated the significance of workplace leadership (Syed et al., 2021). Employees suffered when leadership failed since it played an essential part in the performance of the employees and organizations (Akhtar et al., 2020a). As a result, leaders/leadership play an important role in achieving these goals and encouraging employee performance by rewarding them with their jobs. Similarly, educational institutes in Pakistan face a variety of challenges in terms of infrastructure and resources, teaching, recruitment, and retention, as well as other pressures resulting from rapid technological advancements, increasing demand, a growing need for quality, knowledge diffusion, competitiveness, and globalization (Akhtar et al., 2022).

Responsible leadership (RL) has evolved as a significant topic in organizational studies as a result of the present global financial crisis and CEO wrongdoing (Haque et al., 2019). Maak (2007) defined RL as “*the art and ability involved in building, cultivating and sustaining trustful relationships to different stakeholders, both inside and outside the organization, and in co-ordinating responsible action to achieve a meaningful, commonly shared business vision*” (p. 334). Also, it is important because of protection, acquisition, connection, and understanding (Lawrence and Pirson, 2015). Several studies have recently been published in the leadership literature to explain and comprehend the practice of RL and to investigate its impact on employee outcomes in various sectors (Inam et al., 2021; Javed et al., 2021; Zhang et al., 2021), but very scant in the education sector.

Leaders are great assets to businesses (Coleman, 2007). Responsible executives play an influential job as role models (Akhtar et al., 2020a) for motivating employees (e.g., work engagement). Due to RL, employees are highly motivated and perform beyond their ability (Haque et al., 2019), which results in career success. Therefore, educational institutes need highly responsible leaders to meet the challenges (Miska and Mendenhall, 2018). For instance, RL is concerned with the obligation of inspiring employees for long-term employment in addition to their well-being. Lin et al. (2020) claim that RL encourages employees to take part in decision-making and gives them a feeling of psychological ownership, which supports their intrinsic needs and motivates them to attain greater performance (Lin et al., 2020). Scholars have argued for RL as a means of increasing employee motivation, which can affect trust in leaders (Akhtar et al., 2020a), person–organization fit (Akhtar et al., 2020a), relational social capital (Javed et al., 2021), knowledge sharing (Lin et al., 2020), and employee commitment (Haque et al., 2021).

Drawing upon social information processing (SIP) theory (Salancik and Pfeffer, 1978), we propose and test a model that explains how RL encourages people to engage in career success through job engagement. If an employee believes he or she is a part of the company and has got responsible signals from their leader, they are more prone to perform well. Following SIP theory (Salancik and Pfeffer, 1978), we propose that RL boosts employees career-related success via work engagement (WE).

Salancik and Pfeffer (1978) stated that beyond the influence of individual disposition and characteristics, information cues from the social environment shape human perceptions, attitudes, and behaviors (Salancik and Pfeffer, 1978). Leaders are the best source for the information cues, therefore employees working in the education sector adapt their behaviors according to the informational cues relayed from their organizational leader. Employees in the education sector also adjust their attitudes and behaviors through their interpretation of social situations based on their cognitive processing. As per SIP framework, individuals are particularly attuned to cues from salient sources at the organization, such as RL (Salancik and Pfeffer, 1978). Therefore, if individuals who are working in the education sector experience RL at their workplace then they are more engaged with work, which leads to their career success.

**FIGURE 1:**
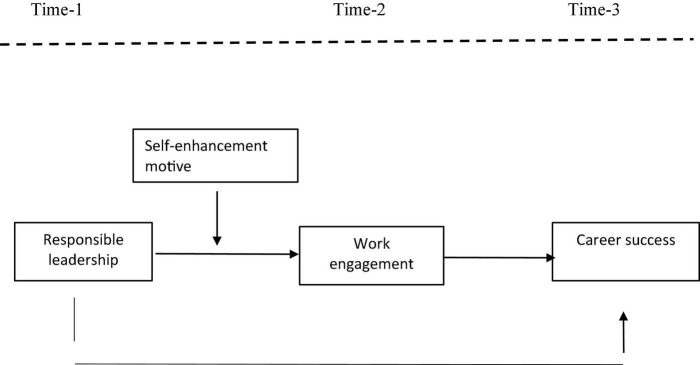
Research model.

In the present study, we specifically focused on the moderating role of self-enhancement motive (SEM) in the relationship between RL and WE. SEM reflects “*an individual employee’s sensitivity to other people’s perception of him/her and his/her level of motivation to adapt his/her behavior in order to project a good self-image to others*” (Yun et al., 2007). Individuals with a high SEM have a strong desire to improve their image in the eyes of others (Yun et al., 2007); hence they are likely to find it interesting to engage at work that promotes their positive image at work. Hence, the present study examines the moderated mediation model by investigating the effect of RL on employee career success via WE at different levels of SEM (see Figure 1).

## Literature Review

### Responsible Leadership and Career Success

Based on the SIP theory, we proposed that RL boosts career success at work in the education sector. Career success is defined as the “accomplishment of desirable work-related outcomes at any point in a person’s work experiences over time” (Arthur et al., 2005). For example, a person who receives many promotions, a rank highest in the hierarchy, and greater pay, but does not feel fulfilled would likely see themselves as unsuccessful (Judge and Bretz, 1994). Therefore, nowadays employees focus on subjective career success, which comprises his/her evaluation related to their career accomplishments with respect to their personal achievement criteria (Gattiker and Larwood, 1988). More directly, “subjective career success may be defined as the individual’s internal apprehension and evaluation of his or her career across any dimensions that are important to that individual” (Van Maanen and Schein, 1977). Leaders have the authority to instruct and assess subordinates’ work performance, which can have a direct impact on the subordinates’ career growth (Astakhova, 2016).

Responsible leadership is an ethical and social-relational phenomenon that extends beyond the dyadic leader–subordinate relationship (Maak and Pless, 2006) and strives for achieving performance objectives (Miska and Mendenhall, 2018). According to SIP theory (Salancik and Pfeffer, 1978), leaders’ responsibility will rub off on their subordinates and subsequently inspire them to take more responsibility. The literature reveals that RL has three main elements (a) effectiveness, employees’ performance has improved as a result of RL (Lin et al., 2020), (b) ethics, RL behaved ethically and lead by example for their followers to act in the right way (Akhtar et al., 2020a), and (c) sustainability, due to focusing more on social, environmental, and economic performance RL leads to sustainability (Javed et al., 2020). RL encourages followers to develop their potential through various methods such as instruction, empowerment, support, participation, equality, communication, and rewards (Maak and Pless, 2006). As a result of these tactics, followers experience more job autonomy and a better feeling at work, which increases internal satisfaction and consequently subjective career success (Pousa and Mathieu, 2015). According to Maak (2007), responsible leaders focus on organizational key characteristics and create a culture that encourages people to achieve common goals. RL prioritizes employee well-being and earns their trust, and workers reciprocate by achieving career success. As a result, we postulated that

H1: RL is positively related to career success.

### Work Engagement as a Mediator

Work engagement is a positive motivating work-related condition in which an employee displays enthusiasm, devotion, and absorption (Schaufeli, 2012). As a result, WE is a situation in which people are completely immersed in their job. Employees who are engaged have a lot of energy, are excited about their jobs, and are frequently so immersed in their work that time seems to fly by Chaudhary and Akhouri (2018), van Dorssen-Boog et al. (2020). Job qualities (e.g., feedback, social support), leadership (e.g., that promotes good effects), and dispositional factors (e.g., conscientiousness) have been identified as common antecedents of WE (Christian et al., 2011).

Responsible leadership is ready to provide workers with learning opportunities so that they may learn and grow (Akhtar et al., 2020a; Javed et al., 2020, 2021). In fact, RL persuades workers to strive toward their objectives by including them in decision making (Maak and Pless, 2006), seeking and respecting their opinion (Lin et al., 2020), and supporting them in times of struggle (Zhang et al., 2021). Employees, in turn, begin to consider the RL as their supporters and exhibit more enthusiasm and devotion to their job. Employees get more engaged since they are sure that performing the task would help them progress. As a result, the argument may be summarized as follows: RL increases employee WE by convincing them that they can achieve their objectives through work. Employees who are engaged are more likely to be involved in career success because they effectively achieve their professional goals and believe they are qualified to perform (Christian et al., 2011).

According to research, when organizations give resources, workers feel WE, which is associated with beneficial outcomes such as organizational commitment (Aboramadan et al., 2019). Job resources have the potential to influence positive outcomes, reflected as career success, not just through reciprocation, but also because when employees feel supported at work, they experience positive feelings. According to the SIP theory (Salancik and Pfeffer, 1978), such informational signals cause a broader range of thinking and acting among employees, such as imagining greater career success.

Work engagement resulting from RL fosters employee career success by broadening employees’ thought and action ranges (Aggarwal et al., 2020). As a result, there is a chance that WE will mediate the influence of RL on career success. In support of the above assumption, Akhtar et al. (2020a) found that the relationship between RL and whistleblowing intents was serially mediated by person–organization fit and trust in leaders in a study of Pakistani workers. Ilkhanizadeh and Karatepe (2017) offered similar data, demonstrating the mediating role of WE in the relationship between CSR practices and career satisfaction. As a result, WE appears to be a viable mechanism for explaining the relationship between RL and professional success. Thus, we proposed that

H2: WE mediates the relation between RL and career success.

### Moderating the Role of Self-Enhancement Motive

In addition to examining the mediating effect of WE on the relationship between RL and career success, it is also necessary to investigate the moderating effect of personal factors (i.e., self-enhancement motives) that might affect the aforementioned relationship. As per SIP, WE is in response to RL. We propose that the availability of personal resources, such as SEM, can increase the influence of accessible resources, in this instance RL. We think that providing a strong positive image or desiring to make a good impression on others can help RL have a stronger impact on WE (Yun et al., 2007).

Self-enhancement is an individual resource that encourages adaptable work practices (Yun et al., 2007). When an individual has a strong desire to create a favorable impression on others, this is referred to as self-improvement motivation (Yun et al., 2007), and in an experienced favorable work environment (i.e., empowerment, training, compensation) this desire is more salient (Choi et al., 2019). Individuals with a high SEM may be more inclined toward WE under RL because of their motivation to achieve a positive self-image. Individuals with a high SEM are sensitive to social perception and have a strong desire to be perceived positively (Yun et al., 2007), hence they excel in their tasks. This desire, we propose, becomes much stronger when people are working in conducive and resource-rich environments (Kwang and Swann, 2010; Choi et al., 2019). The desire or observed reality of seeing oneself in the most positive way is known as self-enhancement (Pfeffer and Fong, 2005). Employees with a high SEM are more aware of how others perceive them and are more motivated to change their behaviors to make a positive impression (Yun et al., 2007). Furthermore, when high SEM employees experience RL, they may wish to steer their organization in a better path to eliminate the source of their negative feelings about their organizational membership and feel more engaged at work (Carter and Guittar, 2014). Employees with a high SEM who are exposed to RL will be even more driven to do their jobs well (Yun et al., 2007). On the other hand, those with a low SEM are less susceptible to external influences (Choi et al., 2019). Individuals with a low SEM are less concerned about their public image. As a result, even after witnessing RL, people will not accomplish their assigned responsibilities with engagement. As a result, we suggest the hypothesis below.

H3: SEM moderates the relationship between RL and WE in such a way that the relationship will be stronger in the case of high SEM or vice versa.

### Indirect Conditional Effect

The above-mentioned mediation and moderation effects, taken together, imply a moderated mediation effect (Preacher et al., 2007). Specifically, the RL is positively and indirectly associated with career success through WE; the level of SEM influences this indirect linkage. Given the importance of career success in boosting organizational effectiveness, employees who are concerned about how others see them may opt to work with thriving, which encourages proactive behavior. Thus, we propose that the positive effect of RL proactive behavior via WE when an employee has a high SEM depicted in Figure 1. Therefore, we predict the following:

H4: SEM moderates the positive and indirect effects of RL on career success through WE such that the indirect effect is stronger when SEM is high.

### Method

The present study aims to investigate the effect of RL on CS via WE at different levels of SEM in the education sector. The present study is of pivotal importance to understand better how to create positive vibes among employees which will be echoed by them within and outside the environment. Data were collected from employees of the education sector.

Each participant has the option of marking each questionnaire with identical codes or any other key of his or her choosing, such as their national identity number, employee number, date of birth, and so on. These codes or keys assisted in the identification of the relevant pair of employee and peer questions. Following that, these keys are eliminated to protect the respondents’ privacy during data submission. Furthermore, it is straightforward to connect the three-time data with the contact person in each organization. Also, the color of the questionnaire, which was white at T1, blue at T2, and green at T3, assisted the responders or the contact person to distinguish each portion from the other. The researcher has tagged each questionnaire with a key of the serial number to ensure that the paired replies of the respondents are from the same individual.

At time 1, we distributed 550 survey questionnaires based on RL and SEM along with demographic details among respondents, and we received 435 usable responses. After a 1-month-interval, we distributed the survey questionnaire of WE among the aforementioned respondents and received 364 usable responses. After a 1-month-interval of time 2, we distributed the survey questionnaire of career success among the aforementioned respondents, and we received 228 questionnaires.

The sample consisted of 164 (72 percent) male and 64 (28 percent) female respondents. Fifty-eight percent of the respondents were married and the rest were single. A total of 105 (46%) respondents had a Master’s degree, 72 (32%) had MPhil/MS degrees, while 51 (22%) had Ph.D. degrees. Fifty-three percent of participants were between the ages of 20 and 30, and 32% were between the ages of 31 and 40. Fifteen percent of participants were above the age of 40. The majority of the participants (57%) had worked for fewer than 5 years. The remainder of the employees were with the company for more than 5 years.

### Variable Measurements

For the measurement of study variables, we used adapted measures. In Pakistan, English is the language of instruction for all high school and university students. It is also the formal means of interpersonal communication at work and is commonly understood by employees in the education industry of Pakistan. Earlier researches utilizing questionnaires in English language in Pakistan have reported no major issues pertaining to language (Akhtar et al., 2020b). For all the constructs in the study as per the research model (Figure 1), we adopted established item scales as used in erstwhile studies. All the items were anchored on a 5-point Likert scale with a range of 1– 5.

***RL:*** To measure RL, we adopted a five-items scale developed by Voegtlin (2011) with the reliability of 0.93. The sample question included, “*My direct leader/manager tries to achieve a consensus among the affected stakeholders*.”

**SEM:** We assessed SEM using a 6-items scale developed by Yun et al. (2007). A sample statement was “I am sensitive to the impression that others have about me”.

**WE:** WE was measured using the nine-items (three-dimension) measure developed by Schaufeli et al. (2006). Sample items included “At my work, I feel bursting with energy”.

**Career success*:*** This construct consisted of five-items taken from Greenhaus et al. (1990). Sample item of the construct included “I am satisfied with the progress I have made toward meeting my goals for the development of new skills.”

### Preliminary Analysis

Since the current study utilized a self-reported survey approach, we applied Harman’s single-factor test to check for the common method bias (CMB). The findings of a single-factor extraction solution with no rotation explained 40.83% of the variation (less than 50%), indicating that CMB is not a major concern for our data set (Podsakoff and Organ, 1986).

## Results

### Conformity Factor Analysis and Correlations

Preceding hypotheses testing, we performed a series of CFAs to verify the convergent and discriminant validity of our study variables. Table 1 reveals that the results of the hypothesized four-factor model (χ^2^/*df* = 2.28; TLI = 0.906, CFI = 0.916, RMSEA = 0.075) fitted the data well, better than the alternative models. The factor-loading ranges are as follows: RL (0.800–0.846), SEM (0.696–0.856), WE (0.510 –0.849), and career success (0.800–0.852). The value of average variance extracted (AVE) of RL (0.68), SEM (0.59), WE (0.58), and career success (0.71) support the variables convergent validity. The discriminant validity that was verified by assessing the √ of each AVE was greater than the correlation between the corresponding variables (see Table 2; Fornell and Larcker, 1981). The findings in Table 3 demonstrated that study variables were correlated with each other.

**TABLE 1:** Confirmatory factor analysis: Validity and reliability.

Latent variables Standardized loadings		Average variance extracted Composite reliability	
*Responsible leadership*		0.68	0.92
RL1	0.800		
RL2	0.828		
RL3	0.846		
RL4	0.821		
RL5	0.839		
*Self-enhancement motives*		0.59	0.89
SEM1	0.696		
SEM2	0.856		
SEM3	0.705		
SEM4	0.804		
SEM5	0.770		
SEM6	0.752		
Work engagement		0.58	0.92
WET1	0.728		
WET2	0.791		
WET3	0.732		
WET4	0.804		
WET5	0.815		
WET6	0.811		
WET7	0.820		
WET8	0.510		
WET9	0.849		
Career success		0.71	0.92
CS1	0.800		
CS2	0.887		
CS3	0.853		
CS4	0.858		
CS5	0.803		

**TABLE 2:** Discriminant validity test results.

Latent constructs	1	2	3	4
1. Responsible leadership	*0.827*			
2. Self-enhancement motives	0.657	*0.766*		
3. Work engagement	0.398	0.462	*0.759*	
4. Career success ‘	0.357	0.261	0.601	*0.807*

**TABLE 3:** Correlations.

Sr #		Mean	SD		2	3	4
1	Responsible leadership (T1)	4.62	1.52	*1*			
2	Self-enhancement motives (T1)	4.89	1.27	0.59**	1		
3	Work engagement (T2)	4.76	1.29	0.44**	0.46**	1	
4	Career success (T3)	4.28	1.53	0.32**	0.23**	0.57**	1

** p < 0.01. SD = standard deviation.

### Hypotheses Testing

Referring to Table 4, the findings disclose that RL has a significant positive relationship with career success (B = 0.14, *p* < 0.001), after controlling for employees’ gender, age, qualification, and organizational tenure, and it supports H1. Referring to Table 4, WE mediates the effect of RL on career success (B = 0.25, CI = 0.17, 0.35), as both the confidence intervals limits did not include zero, which supports H2. Further, the Sobel test (z = 5.84; *p* < 0.001) supported again that WE effect was a mediating variable. Thus, H2 was accepted.

**TABLE 4:** Mediation results.

	M (work engagement)					*Y* (career success)		
	Path	B	SE	P	Path	B	SE	p
RL	A	0.37	0.05	0.00	ć^1^	0.14	0.06	0.02
Work engagement		−	−	−	b^1^	0.66	0.07	0.00
Constant	i^1^	3.05	0.25	0.00	i^2^	0.47	0.33	0.15
		R^2^ = 0.19				R^2^ = 0.40		

**Indirect effect (RL on career success)**								

Indirect Effect via work engagement								
Bootstrap results for indirect effects						0.25 [0.17, 0.35]		
Indirect effect (Sobel Test)						0.25 (z = 5.84)		

*N = 228.*

**FIGURE 2:**
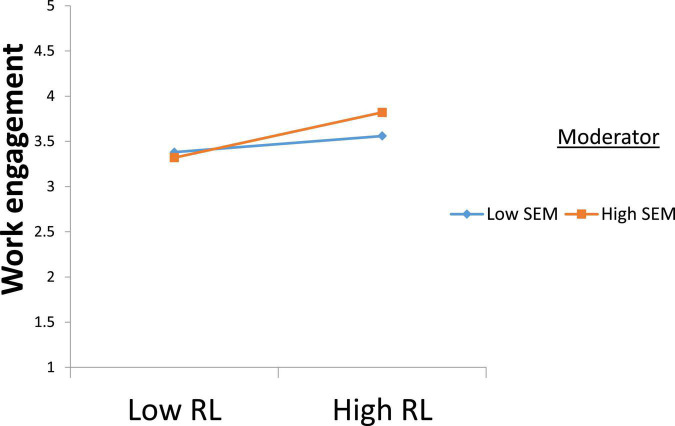
Interaction plot.

As further displayed in Table 5, the interaction term of RL x SEM in predicting WE was significant and positive (B = 0.08, *t* = 2.21, *p* < 0.05). The interactive effect in Figure 2 indicates that RL was more positively linked with WE at high levels of SEM (simple slope = 0.30, *p* < 0.01 95% CI = [0.16, 0.44]) than when it was low (simple slope = 0.10, *p* > 0.05 95% CI = [−0.06, 0.26]). Thus, the H3 effect was supported.

**TABLE 5:** Regression coefficients and conditional indirect effect estimates.

	*M* (work engagement)				*Y* (career success)			
	B	SE	*P*		B	SE	*P*	
RL (X)	−0.18	0.19	0.34		0.14	0.06	0.02	
SEM (W)	0.05	0.14	0.73					
X*W	0.08	0.04	0.03					
Work engagement	−	−	−		0.66	0.07	0.00	
	R^2^ = 0.27				R^2^ = 0.40			

**Moderator**	**Conditional effect of X on M**				**Conditional effect of X on Y via M**			
**SEM**	**B**	**SE**	**LLCI**	**ULCI**	**B**	**SE**	**LLCI**	**ULCI**

SEM − 1 SD	0.10	0.08	−0.06	0.26	0.07	0.07	−0.06	0.21
SEM M	0.20	0.06	0.08	0.32	0.13	0.05	0.04	0.25
SEM + 1 SD	0.30	0.07	0.16	0.44	0.20	0.06	0.08	0.34

*N = 228.*

*B = standardized coefficient, SE = standard error, LLCI = lower limit of confidence interval, ULCI = upper limit of confidence interval.*

We also tested (H4) moderated mediation effect via model 7. Referring to Table 5, findings disclose that the indirect effect was strengthened when SEM was high (B = 0.20, 95% CI = [0.08, 0.34]), whereas indirect effect was weaker and insignificant when SEM was low (B = 0.07, 95% CI = [−0.06, 0.21]). Thus, the H4 effect was supported.

## Discussion

The current study offers a theoretical framework based on SIP theory (Salancik and Pfeffer, 1978), which explains the consequences of RL in the education sector. Recently, researchers invited future studies to investigate the consequences of RL in the education sector (Akhtar et al., 2020a; Javed et al., 2021) with the help of different mediated-moderation mechanisms.

First, the present study finds that when individuals observe RL at work in the education sector then they engage in career success. RL is the most promising in terms of social clues. As it focuses on employees’ personal development by understanding their needs and interest, it cultivates a caring working climate at work which fosters their emotional attachment with the organization (Boiral et al., 2014). Thus, RL provides a positive role model to educate employees, which entices them to engage in positive activities for organizations like career success. These results are consistent with the previous studies which reveal that RL is significantly and positively related to the employee’s positive behavior (Akhtar et al., 2020a).

Second, the present study corroborated that RL significantly affects career success’s via WE. These findings seem logical because responsible leaders in the education sector create an enabling environment and provide required resources to learn and stay vital. In addition, RL develops trustful relations with the employee that help them to engage. Since employees in the education sector learn the behavior expected, rewarded, and punished by RL, as RL builds a trustful relationship with the follower (Akhtar et al., 2020a), a mutually trusted relationship makes the followers feel safe for trial-and-error and learning from past mistakes. So, engaged individuals at work in the education sector actively participate and discuss the ideas related to organizational current and future problems. Thus, they are more likely to engage in career success by sharing the positive information about their education organization to inside and outside stakeholders. Thus, we find that WE mediates the relationship between RL and career success in the education sector. The present study results are aligned with past studies, which revealed that WE mediates the relationships in the education sector (Song et al., 2018; Aboramadan et al., 2019).

Third, findings reveal that SEM moderates the RL and WE relationship in education. In the education sector when individuals feel supported by their organizations, they are likely to thrive more by RL under the SIP theory. The moderating effect of SEM has also been explored by previous researchers (Akhtar et al., 2021a). De Clercq et al. (2021) concluded that SEM buffered the despotic leadership and status gain relationships. Thus, the results of their study are aligned with the present study. Therefore, POS moderates the direct relationship between RL and WE. Furthermore, we find full support for the moderated-mediation argument where SEM positively moderates the indirect link. It reflects that indirect effect was stronger in case of high SEM.

### Theoretical Implications

The present study has several theoretical implications. Firstly, the findings of the present study added value to the literature of RL in the education sector by examining the relationship between RL and outcomes. Only a handful of studies have explored the consequences of RL in the education sector (Freire and Gonçalves, 2021), but no one investigated its consequences in the education sector of Pakistan by using the SIP framework. The current study is unique because it is considered as the preliminary study that examined the impact of RL on career success (internal, external, and online) in the education sector, which is not studied as an outcome of RL. Thus, the researchers examined the employees’ behavioral outcome, i.e., career success, and extended the literature of career success by exploring its new antecedent RL in the education sector.

Secondly, the present study employs the SIP theory (Salancik and Pfeffer, 1978) by contributing to the literature of RL as the previous researchers used role theory, stakeholder theory, and upper-echelon theory to explain RL and the followers’ relationship. By employing this SIP theory, when in the education sector employees observe different social clues from RL, such as trust and information sharing, they may influence the employees’ WE. Further, engaged individuals actively participate in their organizations’ issues and problems. Thus, they are prone to display megaphone by sharing information about the strength and weaknesses of their educational organization. Hence, we added value to the literature by taking WE as an underlying mechanism between RL and career success in the education sector.

Thirdly, the current study explored the consequences of RL and used this construct by proposing a distinctive mediated mechanism and investigating the mediating role of WE in the education sector. The results indicate that the RL is positively related to WE, which further is positively related to career success. WE, in turn, mediates the positive relationship between RL and career success.

Finally, the current study investigated the moderating role of SEM and extends the literature by examining the organizational factor and establishing the link between RL and WE. Additionally, the researcher established the indirect relationship as SEM moderates the mediating relationship between RL and career success through WE.

### Practical Implications

The results of the study have important practical implications for firms working in the education industry. These findings suggest that firms should recruit and develop responsible leaders because they positively influence individual-level outcomes, such as career success. Findings imply that RL brings fortunes to the educational institute because such leadership style helps managers in making employees’ communicative behavior favorable, which consequently improves a firm’s image and reputation. Second, our findings show that responsible leaders can promote career success by developing WE. WE encourages employees to engage in positive megaphoning, for example, WE makes employees feel energized and alive and they tend to be positive accordingly. This suggests that firms should have RL that will promote WE. Results also imply that the firms should brainstorm and map training plans to develop responsible leaders. Third, findings suggest that organizations should practice such initiatives which make their employees believe that they are taken care of, and their contributions are valued and recognized. This belief supports RL to make employees energetic and alive. Further, policymakers should offer incentives to promote RL at the firm-level.

## Limitation and Future Direction

Although the present study provides a novel perspective on RL, it is not without limitations. As we used a time-lagged research design with data from independent sources, our study cannot be characterized as a pure longitudinal design since not all of the study variables were tapped at all the time-periods. Self-reports were used to quantify all variables each time. However, the evidence of discriminant validity, CMB tests, and support for mediation and moderation indicates that this was not the case. Future research can use a comprehensive longitudinal study design, in which all the study variables are assessed at all times. Future studies can build on our existing paradigm by proposing additional processes and contexts under which RL could lead to different outcomes. As positive affectivity and political ineptness could be used as moderators, future studies might use other mediators that might explain how and why RL could result in favorable results for individuals, such as compassion and meaningfulness. The present study investigated the effect of RL on CS via WE by using the SIP theory. Future studies can use different theoretical mechanisms to uncover the consequences of RL, such as the social learning perspective.

## Data Availability Statement

The original contributions presented in the study are included in the article/supplementary material, further inquiries can be directed to the corresponding author/s.

## Ethics Statement

The studies involving human participants were reviewed and approved by COMSATS University Islamabad (CUI), Sahiwal Campus Constitutes Campus Ethics Approval Committee. The patients/participants provided their written informed consent to participate in this study.

## Author Contributions

ML was suggested the idea of this research who wrote the initial protocol of this study. FY performed the statistical analysis of the data. MA collected the data of the study, interpreted them, and wrote the manuscript. All authors reviewed the manuscript and approved its final version.

## Conflict of Interest

The authors declare that the research was conducted in the absence of any commercial or financial relationships that could be construed as a potential conflict of interest.

## Publisher’s Note

All claims expressed in this article are solely those of the authors and do not necessarily represent those of their affiliated organizations, or those of the publisher, the editors and the reviewers. Any product that may be evaluated in this article, or claim that may be made by its manufacturer, is not guaranteed or endorsed by the publisher.
